# Pathophysiological mechanisms underlying early brain injury and delayed cerebral ischemia in the aftermath of aneurysmal subarachnoid hemorrhage: a comprehensive analysis

**DOI:** 10.3389/fneur.2025.1587091

**Published:** 2025-05-23

**Authors:** Hendrik Stragier, Hans Vandersmissen, Sofie Ordies, Steven Thiessen, Dieter Mesotten, Dieter Peuskens, Hugo Ten Cate

**Affiliations:** ^1^Department of Anaesthesiology, Intensive Care Medicine, Emergency Medicine and Pain Therapy, Ziekenhuis Oost-Limburg, Genk, Belgium; ^2^CARIM School for Cardiovascular Diseases, Faculty of Health, Medicine and Life Sciences, Maastricht University, Maastricht, Netherlands; ^3^Faculty of Medicine and Life Sciences, UHasselt, Diepenbeek, Belgium; ^4^Department of Neurosurgery, Ziekenhuis Oost-Limburg, Genk, Belgium; ^5^Thrombosis Expertise Center, Maastricht University Medical Centre, Maastricht, Netherlands; ^6^Center for Thrombosis and Hemostasis, Gutenberg University Medical Center, Mainz, Germany

**Keywords:** aneurysmal subarachnoid hemorrhage, delayed cerebral ischemia, early brain injury, thrombo-inflammation, neuro-inflammation

## Abstract

Early brain injury (EBI) and delayed cerebral ischemia (DCI) are pivotal contributors to morbidity and mortality following aneurysmal subarachnoid hemorrhage (aSAH). Despite advances that have reduced mortality and incidence, aSAH remains a significant public health concern due to its early onset, leading to prolonged periods of diminished quality of life for affected individuals. EBI mechanisms, including endothelial dysfunction, blood–brain barrier disruption, cerebral edema, neuro-inflammation, cortical spreading depolarizations, and oxidative damage, trigger cell death and apoptosis, setting the stage for DCI development in later clinical phases. DCI arises not only from large-vessel vasospasm, but also from other complex pathophysiological processes, including thrombo-inflammation, neuro-inflammation, microcirculatory dysfunction, and glycocalyx disruption. Recognizing and understanding these mechanisms is essential, as early interventions could potentially reduce long-term disability in this population. This comprehensive review offers an in-depth analysis of these pathophysiological mechanisms. As our understanding of these processes continues to evolve, further research is crucial to improving outcomes and reducing the long-term impact of aSAH.

## Introduction

In developed countries, the anticipated lifetime incidence of stroke among adults (aged > 14 years) stands at 5% (1). Within this demographic, 28% manifests as intracerebral hemorrhages, while ischemic strokes account for 62% (2). Subarachnoid hemorrhages, a discrete subtype comprising 2-7% of all strokes, delineates instances where aneurysmal rupture underlies 85% of cases, non-aneurysmal perimesencephalic subarachnoid hemorrhage contribute to 10%, and the residual 5% involves diverse aetiologies such as cerebral venous thrombosis and pituitary apoplexy (3–5).

Globally, the age-adjusted incidence of spontaneous aSAH is 14 cases per 100,000 individuals annually. Of note, there is a substantial elevation in both incidence and proportional frequency of aSAH in low- to middle-income countries compared to their high-income counterparts, a phenomenon predominantly explicable by suboptimal hypertension control. Raised mortality rates in the former settings are likely attributed to deficiencies in management strategies stemming from limited resources and educational constraints (1).

Multiple modifiable and non-modifiable risk factors contribute to aneurysm formation and subsequent aSAH. Modifiable factors encompass smoking, excessive alcohol consumption, insomnia, hypertension and sympathomimetic drug utilization (e.g., cocaine), with smoking and hypertension exerting the most pronounced influence. Non-modifiable risk factors encompass increasing age, family history, prior aSAH, female gender and specific ethnic predispositions (Japanese, Finnish ethnicity) (3–8). Intriguingly, sex-specific incidence differentials become discernible solely in individuals aged 50 and above, a phenomenon likely mediated by hormonal influences. Mitigation efforts targeting modifiable risk factors, particularly smoking and hypertension, have yielded an annual decrement of 0.6% in aSAH incidence over recent decades (4).

Advancements in imaging modalities, endovascular interventions, and enhanced medical management have concomitantly precipitated a reduction in case-fatality following aSAH over time. Nonetheless, this amelioration in fatality is paired with a more modest decline in incidence, underscoring the imperativeness of preemptive interventions. Despite diminished mortality and incidence, aSAH remains a pronounced public health concern, primarily due to its onset at early age, leading to protracted periods of compromised quality of life for afflicted individuals (9, 10). Post-aSAH, the clinical trajectory is characterized by concurrent cerebral injury and multi-system organ dysfunction, demarcated into early and late phases. Notably, next to rebleeding of the ruptured aneurysm, EBI and DCI emerge as prevalent sequelae post-aSAH, wielding substantial influence on compromised functional outcomes and case fatality. This expository review accentuates the nuanced pathophysiological underpinnings of both EBI and DCI and underscores the persistent exigency for further inquiry to delineate refined therapeutic modalities aimed at precluding its occurrence.

## Decoding the early phase post-aSAH: origins of pathophysiology and management of early complications

**Figure 1:**
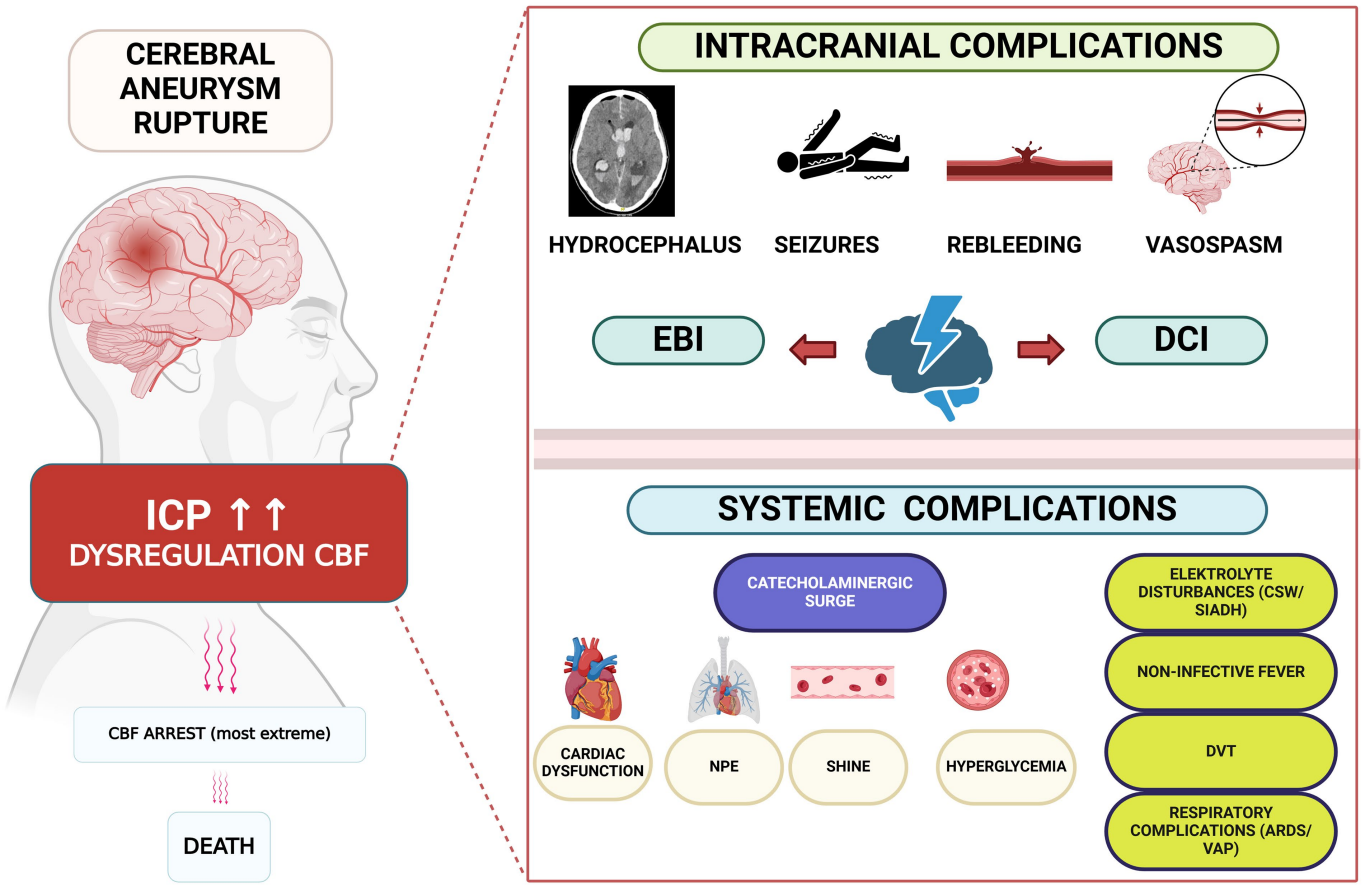
Intracranial and systemic complications following aneurysmal rupture. Created in BioRender. Renette, W. (2025), https://biorender.com/uh0wtez.

In the acute phase subsequent to aSAH, the clinical trajectory is influenced by a confluence of systemic and cerebral determinants. Early identification of these factors is crucial, as they necessitate immediate and targeted interventions (Figure 1). Following aneurysmal rupture, a prompt surge in intracranial pressure (ICP) ensues, precipitating transient global ischemia. The elevation in ICP is a consequence of the mass effect imparted by intracerebral hemorrhage and brain edema, particularly pronounced in cases of poor-grade aSAH (World Federation of Neurosurgical Societies (WFNS) grades 4 and 5). Autoregulatory mechanisms become compromised, resulting in subsequent perturbations of cerebral blood flow. In extremely severe cases this will lead to a cerebral blood flow arrest and subsequent death (9–11).

### Intracranial complications

#### The paradigm of EBI

In juxtaposition to ischemic incidents arising from a precipitous surge in ICP, wherein cerebral blood flow is compromised, the extravasation of blood coupled with subsequent hemoglobin breakdown into noxious substrates initiates pathological processes. Due to its high oxygen demand, the brain is particularly vulnerable to oxidative damage. In a healthy brain, endogenous glutathione-dependent systems, notably involving enzymes such as superoxide dismutase, catalase, and glutathione-peroxidase, are essential for maintaining redox homeostasis. However, after aSAH, various oxidative cascades, driven by increased metabolic requirements and compromised cellular respiration, overwhelm these intrinsic antioxidant defenses, leading to further damage. Organelle dysfunction, especially mitochondrial impairment, becomes a key source of free radicals post-aSAH. Excessive free radicals generated within the mitochondria during ischemia damage mitochondrial proteins, DNA, and lipids. Additionally, mitochondrial reactive oxygen species (ROS) act as potent activators of the nucleotide-binding domain, leucine-rich-containing family, pyrin domain-containing-3 (NLRP3) inflammasome, which may play a role in mediating EBI. Hemoglobin breakdown products further contribute to oxidative stress through the actions of oxyhemoglobin and iron, generating superoxide and hydrogen peroxide. These breakdown products also activate neuro-inflammatory pathways, particularly the Toll-Like receptor 4 (TLR-4) activated NFκB pathway (11).

These intricate cascades eventuate in the disruption of the blood–brain barrier, microcirculatory dysfunction, and the manifestation of cerebral edema. In aggregate, these sequelae precipitate neuronal cell demise and concomitant neurological dysfunction, collectively encapsulated within the paradigm of EBI (11).

#### The critical hours

Next, to EBI, which will be discussed extensively later on, different other intracranial complications have an important impact on the clinical trajectory (5). Acute hydrocephalus manifests in 12-31% of aSAH patients, worsening cerebral hemodynamics. Urgent intervention involves external ventricular drainage placement, a procedure associated with a 15% incidence of tract hemorrhage, albeit mostly clinically inconsequential (9). The management of acute ICP elevation involves administration of hypertonic saline or mannitol, both demonstrating efficacy. Nevertheless, the optimal dosage and treatment strategy for enhancing outcomes necessitate precise delineation (5, 12).

A well-recognized complication of aSAH, critically affecting patient outcomes, is rebleeding, typically occurring within the first 72 h, with the highest risk during the initial 24 h (incidence of 3-6% < 24 h) (5). Consequently, it is recommended to treat patients expeditiously (preferably within 24 h) to secure the aneurysm using either endovascular treatment or surgical clipping. Nevertheless, current research has not shown a clear benefit of treatment within the first 24 h compared to treatment within the 24-72 h window (5, 13). In the later phases following aSAH, DCI, seizures, subacute and chronic hydrocephalus are key determinants of case fatality and functional outcomes (5, 14).

### Systemic complications

In addition to cerebral factors, the patient’s clinical trajectory will be significantly influenced by the occurrence of systemic complications (14).

#### A catecholaminergic surge

During the acute phase post-aSAH, a transient global ischemia induces a “catecholaminergic surge” due to a hypothalamic ischemic insult and brain stem compression. Elevated sympatico-adrenal activation results in endothelial perturbation, aligning with the paradigm of shock-induced endotheliopathy (SHINE). Concomitant hypoperfusion and central nervous injury amplify hypothalamic-hypopituitary-adrenal axis activity, yielding heightened plasma endocrine catecholamines and direct sympathetic outflow with neurotransmitter catecholamine release from the nervi vasorum. These mechanisms collectively contribute to endothelial compromise, capillary permeability, and microvascular thrombosis (15, 16). Consequently, ischemic events during the early phases of aSAH incite vascular dysfunction, culminating in EBI and DCI (15). Transient global ischemia concurrently disrupts the blood–brain barrier, induces endothelial injury, vasogenic edema, and portends adverse clinical outcomes (17).

#### Stress cardiomyopathy

The aforementioned catecholaminergic surge not only elicits endothelial dysfunction and microthrombosis but also begets systemic manifestations, substantially amplifying morbidity and mortality in aSAH cohorts. Prominent systemic manifestations encompass cardiac dysfunction, characterized by wall motion abnormalities, biomarker elevations, or electrocardiographic aberrations, correlating with an augmented risk of unfavorable outcomes and DCI. Stress cardiomyopathy (“Tako Tsubo”), prevalent in cases of poor-grade aSAH, may necessitate substantial vasopressor and inotropic support, potentially escalating to intra-aortic balloon pump therapy (9, 10, 16).

#### Neurogenic pulmonary edema

A commonly recognized complication during the acute phase of aSAH resulting from heightened catecholaminergic activity, particularly in cases of high-grade aSAH, is neurogenic pulmonary edema, contributing to respiratory impairment. In the subsequent stages of the clinical progression, this respiratory dysfunction may be exacerbated by the onset of acute respiratory distress syndrome, cardiac dysfunction, fluid overload and ventilator-associated pneumonia (5, 9, 10).

#### Elektrolyte disorders

Disorders of electrolytes are common among patients suffering from aSAH: hypokalemia, hypophosphatemia, hypomagnesemia, due to renal losses. Also, sodium disorders with both hyponatremia, as a result of cerebral salt wasting (CSW), syndrome of inappropriate antidiuretic hormone (SIADH) and hypernatremia, resulting from diabetes insipidus, are common (5).

## Early brain injury (≤72 h)

Within the critical initial 72 h post-aSAH, the onset of EBI is plausible, and its manifestation bears a substantial association with an increased risk of adverse outcomes (9). Despite the severity of immediate consequences following hemorrhage, EBI introduces complexities into the clinical trajectory, significantly contributing to enduring neurological deficits.

Following the rupture of an aneurysm, a surge in ICP ensues, culminating in transient global ischemia and cerebral edema. Initially, the determination of EBI relied upon a dichotomous assessment predicated on the existence of global cerebral edema. Using this approach, 20% of patients exhibited EBI (8% upon admission, 12% shortly thereafter). In more recent times, the advent of semiquantitative methodologies such as “subarachnoid hemorrhage early brain edema score,” a score system based on early changes in clinically obtained computed tomography, and quantitative methodologies (selective sulcal volume, automated or not), have yielded heightened EBI identification rates (37 to 61% of patients with aSAH) (11, 18–20).

The imperative nature of identification lies in its robust association with an escalated risk of unfavorable outcomes post-aSAH (9).

The initial pathological consequences of aneurysmal rupture, namely, the extravasation of haem-laden blood and its toxic substrates, the abrupt elevation of ICP, impaired cerebral perfusion, and dysregulation of cerebral autoregulation culminating in cerebral edema, serve as triggers for a cascade of secondary pathophysiological mechanisms that underlie the development of EBI.

Distinct mechanisms can be discerned within the pathophysiology of EBI, encompassing cerebral metabolic distress (nitric oxide uncoupling, oxidative stress, and adenosine triphosphate (ATP) depletion), cortical spreading depolarization, neuro-inflammation (microglial activation, release of cytokines and chemokines, and adhesion molecule expression), microvascular dysfunction, activation of the coagulation system (platelet activation and aggregation, microthrombi formation), and cellular phenomena such as endothelial cell apoptosis, autophagy, neuronal swelling and cell death (5, 9–11, 21, 22).

Over time, blood accumulation within the subarachnoid space exacerbates and modulates the pathophysiological mechanisms that germinate during the early phase. Consequently, these mechanisms furnish a substrate upon which DCI may ensue during the later phase of the clinical course, typically spanning from day 3 to day 14 (9–11, 17).

### Early microvascular dysfunction: the role of endothelial dysfunction

Following aSAH, a series of deleterious pathways compromise cerebral homeostasis. Early microvascular dysfunction emerges as pivotal contributor to this disruption, involving both smooth muscle cells and endothelial cells (11).

The endothelial contribution to microvascular dysfunction is mediated by two primary mechanisms: the formation of microthrombi associated with thrombo-inflammation and a significant perturbation of the nitric oxide (NO)-cyclic guanosine monophosphate (cGMP) signaling pathway (23, 24). NO is integral to the regulation of smooth muscle cell relaxation and maintenance of various endothelial cell functions, including survival, proliferation, anti-inflammatory signaling, tight junction integrity, and platelet activation. It is synthesized from L-arginine via nitric oxide synthetase (NOS), with endothelial NOS (eNOS) being critical for sustaining baseline stability and tone of cerebral microvasculature. In the context of aSAH, the degradation of extravasated blood releases free haem molecules, which adversely affect NO bioavailability. This occurs through two mechanisms: a reduction in NO production by NOS and the sequestration of NO by haem, due to its high-affinity interaction with iron (11, 22–27). Moreover, intracellular calcium signaling pathways are implicated in the microvascular dysfunction that contributes to EBI. In the acute phase following aSAH, there is a marked increase in intracellular Ca^2+^ concentrations within both endothelial cells and smooth muscle cells, leading to cerebral vasospasm (28). Recent evidence highlights the role of L-type voltage dependent calcium channels in Ca^2+^-mediated pressure-dependent vasoconstriction, which contributes to the neurological deficits characteristic of EBI (17).

Additionally, the NO-cGMP pathway is critically involved in microthrombus formation via thrombo-inflammation, which is also a key driver of DCI (11, 21, 23, 24).

### Cortical spreading depolarization

Cortical spreading depolarizations (CSD) are waves of depolarization that traverse the cerebral cortex following various types of brain injury, such as ischemic and hemorrhagic stroke, aSAH, and traumatic brain injury. These depolarizations are observed in 50-90% of patients with these conditions and play a pivotal role in the pathophysiology of both EBI and DCI following aSAH (29).

CSDs can initiate immediately after the onset of brain ischemia due to aneurysmal rupture and represent a primary mechanism of electromechanical membrane failure. This failure leads to neuronal swelling, disruption of dendritic architecture, and excessive neurotransmitter release, culminating in neurotransmitter-induced excitotoxicity (10, 29). Under conditions of severe ischemia, ATP depletion causes the failure of the Na^+^/K^+^ pump, the principal ion transporter responsible for maintaining transmembrane cation gradients. This pump failure triggers a depolarization wave that radiates outward from the site of origin. Concurrently, this phenomenon induces a spreading depression of electrocorticographic activity, an increase in metabolic demand, and substantial disturbances in ionic homeostasis (10, 30).

A key mechanism underlying the propagation of CSDs involves the passive diffusion of extracellular K^+^ and glutamate, which induces depolarization within the cortical grey matter (17). A positive feedback loop, driven by interactions with N-methyl-D-aspartate (NMDA) receptors, Ca^2+^ channels, and other voltage-gated channels, exacerbates glutamate release and K^+^-efflux, thereby sustaining the waves of depolarization (17). This process is characterized by the release of large quantities of neurotransmitters following depolarization, which can intensify neurotransmitter-induced excitotoxicity through activation of NMDA and *α*-amino-3-hydroxy-5-methyl-4-isoxazolepropionic acid (AMPA) receptors, leading to excessive neuronal excitation and eventual cell death (10, 31).

CSDs can also propagate into brain regions initially unaffected by the triggering event, inducing a loss of spontaneous and evoked activity in neighboring neural networks, a phenomenon referred to as “spreading depression” (29, 32).

In patients with aSAH, CSDs contribute to dynamic alterations in cerebral blood flow. In healthy tissue, a depolarization wave increases metabolic demand, eliciting a reactive hyperemic response. However, in compromised tissue, where NO bioavailability is diminished, due to NO scavenging, inhibition of eNOS by hemolytic products, and CSD-induced suppression of NO production, neurovascular coupling becomes disrupted. Consequently, metabolic demands are unmet, leading to sustained hypoperfusion, an inverse ischemic response, and the onset of new spreading ischemia. In the later stages of aSAH, these CSDs can precipitate DCI (10, 17, 29, 30, 33, 34).

Additionally, CSDs are implicated in the exacerbation of neuroinflammatory processes and the formation of microthrombi, further contributing to the pathogenesis of DCI (30, 34).

### Disruption of blood brain barrier

Another key factor in contributing to EBI following aSAH is the disruption of the blood–brain barrier (BBB). Increased BBB permeability is observed in patients with aSAH who experience poor outcomes, and early assessment of BBB integrity using extended-pass perfusion computed tomography or automated magnetic resonance imaging, however difficult to perform, may provide valuable prognostic information, guiding clinical management in these cases (35–38).

The BBB is constituted by microvascular endothelial cells interconnected by tight and adherent junctions, encased by pericytes and astrocytes, which envelop the cerebral capillaries. This barrier is essential for maintaining cerebral homeostasis and autoregulation, including nutrient delivery, ion balance, neurotransmitter regulation, restriction of plasma molecule entry, and protection against neurotoxins (39). Additionally, the BBB regulates immune cell infiltration into the central nervous system (CNS) during inflammation (11, 39).

In the very early phases following aneurysmal rupture, there is a profound disruption of the BBB, allowing toxins and leukocytes to infiltrate the CNS, which triggers significant inflammatory and oxidative cascades (11). This disruption is rapid, occurring within 30 min post-rupture, peaking at 3 h, with a secondary peak of injury at 72 h (35). During this acute phase, transient global ischemia induces rapid apoptosis of endothelial cells, along with perivascular pericytes and astrocytes, leading to an immediate compromise of the BBB’s structural integrity. Additionally, toxic byproducts of blood degradation like oxyhemoglobin and iron, further destabilize the BBB (with failure to exert its neuroprotective functions) by promoting the production of inflammatory cytokines and destructive enzymes, exacerbating EBI and contributing to the development of DCI (35).

Several factors contribute to endothelial cell apoptosis shortly after aneurysmal rupture, including oxidative stress, oxyhemoglobin, and iron overload. Oxyhemoglobin induces cytotoxicity in endothelial cells by activating caspases (3, 8, 9) and matrix metalloproteinase (MMP)-9 (35). The introduction of blood into the subarachnoid space, ventricles, and brain parenchyma activates multiple molecular pathways. Blood components stimulate toll-like receptor 4 (TLR-4), initiating the nuclear factor kappa B (NFκB) pathway and driving the transcription of proinflammatory cytokines, including tumor necrosis factor-*α* (TNF-α), interleukin (IL)-1β, IL-6, IL-8, and IL-12. NFκB, along with other inflammatory cytokines, upregulates MMP-9, which degrades zonula occludens-1, compromising tight junction integrity and accelerating BBB breakdown (11, 35). MMP-9 further degrades crucial extracellular matrix components, such as collagen IV, fibronectin, and laminin, contributing to the continued deterioration of the BBB. Proinflammatory mediators, including IL-1β, IL-6, IL-8, and IL-12, also promote endothelial cell apoptosis and upregulate specific cell adhesion molecules, facilitating the migration of macrophages and neutrophils into the subarachnoid space. This infiltration leads to the release of additional proinflammatory substances, further exacerbating BBB disruption (11, 35, 39). Endothelial cells are highly vulnerable to oxidative stress, and the excessive production of free radicals, alongside toxic byproducts of erythrolysis and iron overload, causes significant damage, leading to apoptosis and further compromise of the BBB.

### Cerebral edema

Emerging evidence indicates that cerebral edema plays a crucial role in the pathogenesis of EBI following aSAH. The presence of cerebral edema is strongly associated with poor clinical outcomes, underscoring its significance in the pathophysiology of EBI (11, 40, 41).

The development of cerebral edema post-aSAH involves multiple mechanisms, with a distinction between vasogenic and cytotoxic edema. Cytotoxic edema results from failure of cellular homeostasis due to transient global ischemia and ischemic energy failure, leading to osmotic shifts and the dysfunction of membrane ion pumps, such as aquaporin-4 (AQP4). AQP4 are water-specific channels that allow passive water diffusion across cell membranes in response to osmotic gradients. These channels can exacerbate cerebral edema by increasing membrane permeability. However, the role of AQP4 in aSAH-related edema is complex and not fully understood, as current evidence presents conflicting findings (41). It is important to note that downstream pathways may amplify cytotoxic edema, with microglial activation during neuro-inflammation in EBI causing disorganization of AQP4 channels in astrocytes, further aggravating edema.

Vasogenic edema, closely linked to BBB disruption, arises from endothelial cell dysfunction, breakdown of tight junctions, and degradation of the basal lamina (11, 41). Ischemic insult leads to the upregulation of vascular endothelial growth factor (VEGF), which promotes the formation of capillary fenestrations, thereby facilitating the development of vasogenic edema (41).

### Neuro-inflammation

Neuro-inflammation is a central component in the pathophysiology of EBI. Moreover, platelet activation has been associated with EBI following aSAH. Notably, increased platelet activation and inflammation correlate with more severe injury, as indicated by higher scores on the Hunt & Hess scale, and are linked to worse outcomes in EBI (42).

In the immediate aftermath of aSAH, there is a robust activation of various immunological pathways. The infiltration of leukocytes and the activation of resident microglia form the foundation upon which subsequent events unfold. One of the earliest responses to aSAH is massive infiltration of the CNS by leukocytes, particularly neutrophils, which activate the innate immune response and stimulate resident microglia. This activation triggers neuroinflammatory pathways, leading to the recruitment of additional neutrophils, monocytes, and other immune cells, thereby amplifying the inflammatory response. Consequently, the early post-aSAH phase is marked by a pronounced proinflammatory response, which has both acute and long-term detrimental clinical effects (11, 43). A recent animal study explored the role of perivascular Neutrophil Extracellular Traps (NET) in the development of microvasospasm following experimental aSAH (44). NETs are composed of DNA-histone complexes and various antimicrobial proteins released by activated neutrophils. While these structures are effective in capturing and neutralizing pathogens, they can also cause damage to host cells under pathological conditions. Nakagawa et al. demonstrated that after injecting blood into the prechiasmatic cistern of mice, there was a significant neutrophil infiltration into the perivascular space, accompanied by substantial release of NETs adjacent to arterioles, which correlated with the occurrence of microvasospasms. Furthermore, both neutrophil depletion (via administration of neutrophil-specific antibodies) and the enzymatic degradation of perivascular NETs (via intracisternal DNase treatment) alleviated micro vasospasms (44). These findings suggest that targeting perivascular NETs may represent a novel therapeutic strategy for preventing microvasospasms. Another animal study revealed that intravascular NETs formed following experimental aSAH contribute to vascular occlusion. In this model, depletion of neutrophils using neutrophil-specific antibodies and inhibition of NET formation through a peptidylarginine deiminase 4 (PAD4) inhibitor both effectively prevented DCI. By contrast, enzymatic degradation of NETs with DNase-I led to only marginal improvement in outcomes. This study also investigated plasma biomarkers of NET formation, neutrophil elastase and citrullinated histone H3, in human patients with aSAH (45). Following aSAH, plasma levels of these markers rose dramatically, indicating a rapid neutrophil response and NET formation occurring within hours of aSAH onset and persisting for at least 10 days. Importantly, patients who developed DCI exhibited significantly higher plasma levels of neutrophil elastase, whereas citrullinated histone H3 levels showed no significant difference between patients with and without DCI during the 10-day observation period (45). On top, an animal study showed a robust correlation between NETs and early microthrombosis after experimental aSAH (46).

As previously discussed, toxic blood components interact with TLR-4, inducing the production of various proinflammatory cytokines (e.g., IL-1β, IL-6, IL-10, TNFα) via the NFκB pathway, involving both resident CNS cells and infiltrating leukocytes. TLR-4 is highly expressed in microglia, astrocytes, and endothelial cells within the CNS, underscoring its role in the inflammatory response. Neuro-inflammation also contributes to the development of DCI (11, 24).

The critical role of microglia in neuro-inflammation during EBI is well established. Microglial activation in EBI is perpetuated by ongoing exposure to toxic blood breakdown products, such as iron and haem degradation byproducts. This sustained exposure is primarily driven by congestion within the meningeal lymphatic system (47).

Despite their proinflammatory role (contributing to BBB disruption, neuro-inflammation, cerebral edema, microglial inflammation), microglia also engage in protective functions during EBI, particularly in scavenging hemoglobin via the CD163 pathway (hemoglobin-haptoglobin scavenger). This process, while initially mediated by microglia, is supplemented by peripheral macrophages, which are more effective at hemoglobin breakdown. In addition to these roles, microglia are implicated in other processes during EBI, such as white matter injury, demyelination, and glutamate excitotoxicity, which leads to neuronal and glial cell death. These findings highlight the complex and multifaceted roles of microglia in both exacerbating and mitigating injury during the EBI phase (47).

### Oxidative cascades

The CNS’s high demand for oxygen renders neuronal cells exceptionally susceptible to oxidative damage. These cells, owing to their elevated metabolic activity, produce substantial amounts of free radicals as a natural consequence of cellular respiration. To counterbalance this, the brain relies on its intrinsic antioxidant defence mechanisms, particularly the glutathione pathway. However, in patients with aSAH, a significant imbalance arises between metabolic demand and cellular respiration, leading to an excessive accumulation of toxic free radicals that overwhelm the brain’s endogenous antioxidant systems, resulting in severe oxidative damage (11, 24).

Following aSAH, a predominant source of ROS is the oxidative reactions involving various haem groups released during erythrolysis within the subarachnoid space. The degradation of hemoglobin, especially through the oxidative processes of oxyhemoglobin and iron, generate free radicals, such as superoxide and hydrogen peroxide (11, 24, 41). Oxyhemoglobin contributes to the disruption of the BBB and triggers neuro-inflammation, while iron itself is particularly deleterious, as it can accumulate in brain tissue, leading to cerebral edema and neuronal death during EBI. Free iron reacts with hydrogen peroxide and superoxide to produce hydroxyl radicals, highly reactive species capable of damaging different biomolecules. In the brain, most non-haem iron exists in the ferric form (Fe^3+^), sequestered within ferritin. Iron can only be released after reduction to its ferrous form (Fe^2+^), a process facilitated by superoxide radicals, acidic pH, and catecholamines, conditions prevalent during hypoxia and ischemia (48, 49). Elevated intracellular iron accumulation has been linked to iron-mediated neurotoxicity, as the saturation of regulatory mechanisms responsible for maintaining iron homeostasis can result in severe neurotoxicity. This manifests through iron-induced oxidative stress, brain edema, and neuronal cell death (49).

### Cell death and apoptosis

The pathophysiology of EBI encompasses a range of mechanisms, such as BBB disruption, oxidative stress, neuro-inflammation, and cerebral edema, which converge to cause neuronal cell death. Different mechanisms of neuronal cell death are present in EBI, including apoptosis, pyroptosis, ferroptosis, and necroptosis (11).

The mechanisms underlying neuronal cell death in EBI are diverse and have been extensively studied. Apoptosis, one of the earliest forms of neuronal cell death, occurs soon after the initial insult. This process can be triggered by intrinsic pathways, including caspase activation and mitochondrial cytochrome C release, as well as by extrinsic pathways, such as TNFα receptor engagement (11).

Another critical mechanism in EBI is autophagy, a process essential for neuronal survival that recycles damaged organelles and cellular debris following ischemic injury (11).

Pyroptosis is a form of inflammatory cell death that is critically dependent on the activation of the NLRP3 inflammasome. This process involves an initial priming step, in which damage-associated molecular patterns (DAMPs), recognized by TLR-4, stimulate the expression of IL-1β, IL-18, and NLRP3 through the activation of NFκB pathway. In a subsequent step, the activation and assembly of the inflammasome are triggered by various upstream signals, including mitochondrial dysfunction, mitochondrial ROS production, the release of mitochondrial DNA, lysosomal rupture, potassium and chloride efflux, sodium influx, and additional DAMPs. Upon activation of NLRP3, the apoptosis-associated speck-like protein containing a CARD (ASC) and caspase-1 are recruited to assemble the inflammasome complex. Oligomerization of the NLRP3 complex leads to the activation of caspase-1, which subsequently promotes the maturation of the proinflammatory cytokines IL-1β and IL-18. Furthermore, activated caspase-1 (CASP1) cleaves gasdermin D (GSDMD) into its N-terminal fragment (N-GSDMD), which forms membrane pores and initiates pyroptosis. Caspase-8 and caspase-3 also contribute by cleaving gasdermin E (GSDME), resulting in additional pore formation and cytokine release (50). The critical role of the NLRP3 inflammasome will be further explored in subsequent sections.

As previously discussed, disturbances in iron metabolism play a critical role the pathophysiology following aSAH, with abnormal increases in intracellular ferrous iron serving as a key initiating factor of ferroptosis (51). Ferroptosis is an iron-dependent form of programmed cell death characterized by excessive lipid peroxidation (11, 51). Its distinct morphological features include mitochondrial shrinkage, loss of mitochondrial cristae, and increased membrane density. Biochemically, ferroptosis is marked by the inability to reduce excessive lipid peroxides and the accumulation of large amount of ROS within the membrane lipids, thereby impairing the fundamental biological functions of the cell membrane and ultimately leading to cell death. This process is further accompanied by depletion of intracellular glutathione and loss of glutathione peroxidase 4 activity. Lipid peroxidation is widely regarded as a hallmark of ferroptotic cell death (52).

## Pathophysiology of delayed cerebral ischemia

DCI is defined as the occurrence of a focal neurological impairment (e.g., hemiparesis, aphasia, apraxia, neglect), or a decrease of at least 2 points on the Glasgow Coma Scale (total scale or one of the components) lasting for at least one hour which was not apparent immediately after aneurysm occlusion and which cannot be attributed to other causes (clinical assessment, radiographic findings of the brain, appropriate laboratory studies) (53).

DCI following aSAH is a major contributor to poor neurological outcome and typically will affect 20-30% of aSAH survivors (43).

### Beyond vasospasm

For decades, DCI was attributed primarily to cerebral vasospasm. However, different clinical trials targeting these cerebral vasospasms did not significantly improve outcomes. Moreover, emerging evidence from different kind of studies, suggests that DCI has a multifactorial etiology. Notably, while approximately 70% of patients, as shown by radiographic findings, will develop cerebral vasospasm following aSAH, only 20-30% experience DCI (54). Furthermore, DCI can occur in the absence of vasospasm and may affect brain regions distant from sites of vasospasm (23).

Given the assumption that DCI after aSAH was mediated by vasospasm in large and medium-sized intracranial arteries, various therapeutic interventions targeting this mechanism have been explored. For instance, the CONSCIOUS-1 trial demonstrated that Clazosentan, an endothelin receptor antagonist, significantly and dose-dependently reduced moderate to severe angiographic vasospasm following aSAH (55). However, in the subsequent CONSCIOUS-2 trial, administering Clazosentan at 5 mg/h for 14 days post-aSAH treated with surgical clipping did not significantly impact mortality, vasospasm-related morbidity, or functional outcomes (56). Similarly, the CONSCIOUS-3 trial, which was prematurely halted, evaluated Clazosentan following endovascular treatment of aSAH. Although it showed a significant reduction in vasospasm-related morbidity and all-cause mortality, there was no improvement in outcomes measured by the extended Glasgow Outcome Scale (GOS) (57). These trials underscored the discrepancy between reducing angiographic vasospasm and achieving meaningful clinical improvement. Side effects of Clazosentan, such as increased incidence of hypotension, anemia, and pulmonary complications observed in CONSCIOUS-1, might partially explain this disconnect (55, 58).

In contrast, more recent data from two Japanese double-blind, placebo-controlled phase 3-trials, one involving endovascular coiling, and the other surgical clipping, demonstrated that Clazosentan (10 mg/h) significantly reduced the combined incidence of vasospasm-related morbidity and all-cause mortality in patients post-aSAH (59). The REACT trial sought to further assess the impact of Clazosentan, when added to standard care, on clinical deterioration resulting from DCI following aSAH. However, Clazosentan, administered at 15 mg/h for up to 14 days, did not demonstrate a significant effect on preventing clinical deterioration due to DCI (60, 61). Discrepancies among clinical trials evaluating vasospasm treatments following aSAH largely stem from methodological heterogeneity, including underpowered and single-center designs, diverse patient populations, and inconsistent control of confounding factors. Variability in outcome measures, ranging from angiographic to clinical vasospasm, and from DCI to functional outcomes, further complicates interpretation, as these endpoints differ in their sensitivity and specificity to vasospasm. Differences in treatment timing, dosing regimens, and inclusion criteria also contribute to inconsistent findings. While some interventions demonstrate radiographic improvement in vasospasm, this has not reliably translated into enhanced neurological outcomes. Additionally, the multifaceted pathophysiology of aSAH, in which vasospasm constitutes only one of several contributors to DCI, further limits the ability to isolate the therapeutic impact of vasospasm-targeted treatments.

**Figure 2:**
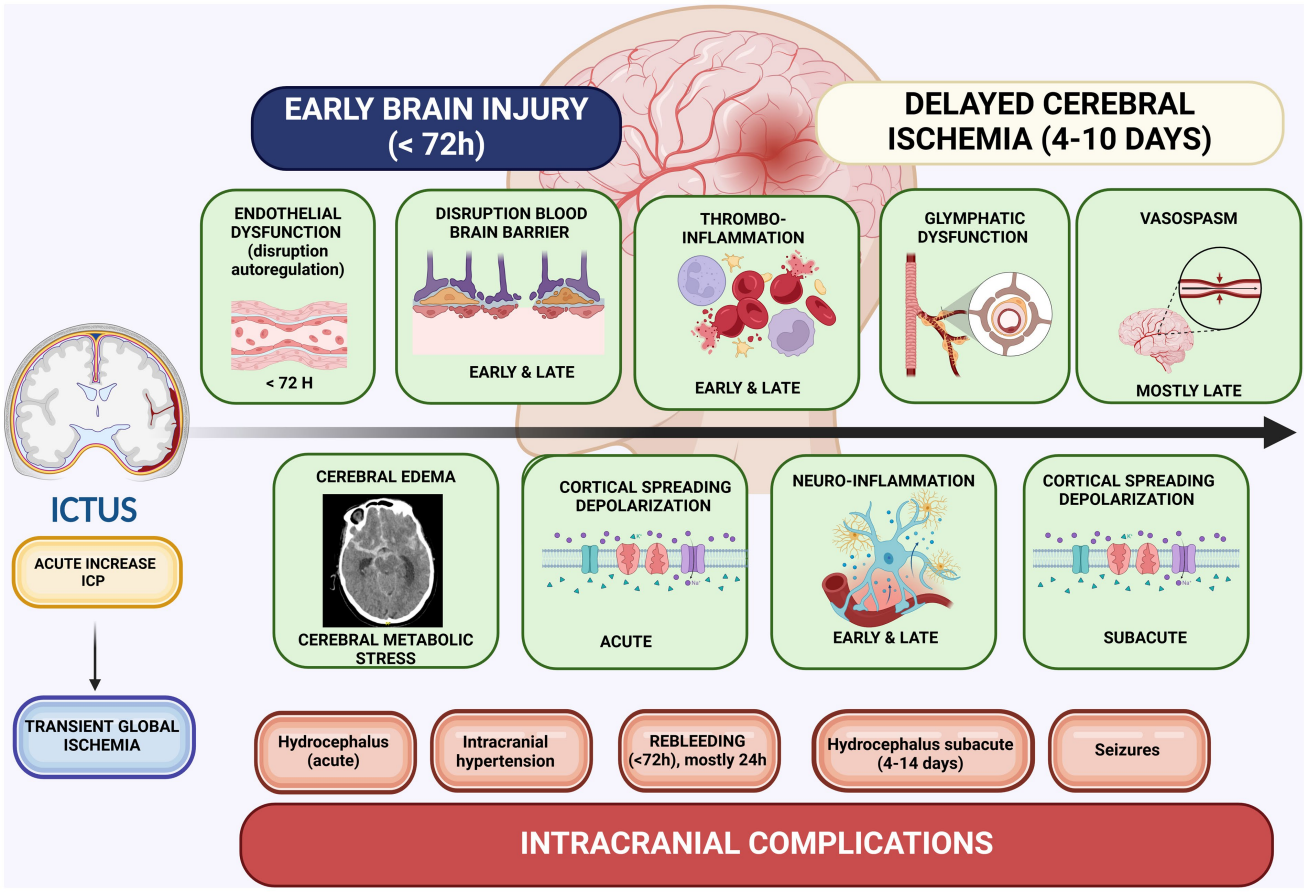
Pathophysiological mechanisms in EBI and DCI. Created in BioRender. Renette, W. (2025) https://BioRender.com/r52x457.

To date, nimodipine is the most effective treatment for reducing morbidity and mortality following aSAH (12, 62, 63). A recent network meta-analysis comparing various therapeutic agents found that nimodipine significantly reduced all-cause mortality, cerebral vasospasm, DCI, and disability, as measured by the GOS and modified Rankin Scale (42). Notably, by blocking voltage-dependent L-type calcium channels, nimodipine’s beneficial effects are likely mediated through mechanisms other than vasospasm reduction: inhibition of CSDs, antithrombotic effects (diminished platelet aggregation and increased endogenous fibrinolysis), anti-inflammatory properties, and some neuroprotective effects (less endothelin neurotoxicity, less intracellular swelling, less oxygen free radicals, and less intracellular Ca^2+^-influx) (12, 64, 65). Indeed, vasospasm is not the sole contributor to DCI. The underlying pathophysiology involves a complex interplay of microcirculatory dysfunction, thrombo-inflammation, neuro-inflammation, and spreading depolarizations (33, 58) (Figure 2).

### Glycocalyx disruption, platelet activation, and nitric oxide pathway dysfunction in the pathophysiology of delayed cerebral ischemia

In a small case series, DCI was associated with significant elevations of soluble markers indicative of endothelial injury, including vascular cell adhesion molecule 1 (VCAM-1) and other cell adhesion molecules, along with increased levels of syndecan-1, a specific biomarker of glycocalyx degradation. Furthermore, elevated concentrations of MMPs, enzymes known to degrade glycocalyx components, were detected in both cerebrospinal fluid (CSF) and circulating blood. These findings support the hypothesis that glycocalyx disruption contributes to the pathophysiology of DCI through mechanisms such as neuro-inflammation, microthrombosis, endothelial dysfunction, and oxidative stress (66). However, the precise molecular mechanisms underlying glycocalyx degradation remain incompletely elucidated. Current evidence suggests that the release of disintegrins and MMPs in response to inflammation and ischemia plays a central role, and that enhanced activity of heparinase and hyaluronidase further contributes to glycocalyx breakdown (67).

The endothelial glycocalyx is a critical component of cerebral vascular homeostasis, functioning as a dynamic barrier that regulates vascular permeability and neuroinflammatory signaling. Closely associated with the BBB and the neurovascular unit, the glycocalyx modulates intercellular interactions and serves as a major antioxidative defence system (67). Structurally, it consists of glycoproteins and proteoglycans linked to glycosaminoglycans, and undergoes continuous renewal in response to hemodynamic forces such as shear stress. By preventing the adhesion of plasma proteins and circulating cells to the endothelial surface, the glycocalyx maintains endothelial quiescence and inhibits the initiation of inflammatory and thrombotic cascades (67, 68). Disruption of this structure exposes the endothelium to circulating leukocytes and platelets, triggering an inflammatory response that exacerbates neuro-inflammation and microvascular thrombosis. Furthermore, glycocalyx degradation impairs its ability to bind key anticoagulant proteins, such as activated protein C (aPC), tissue factor pathway inhibitor (TFPI), and antithrombin III, thereby shifting the hemostatic balance toward a procoagulant state.

In addition to its barrier and anticoagulant functions, the glycocalyx plays a pivotal role in regulating platelet activation. Under physiological conditions, it sequesters von Willebrand factor (VWF), a multimeric glycoprotein produced and stored in endothelial cells. Upon secretion, VWF undergoes shear stress-induced conformational changes that expose its A1 domain, facilitating platelet adhesion via interaction with platelet surface receptors. Electrostatic interactions between the heparin-binding site of VWF A1 domain and the negatively charged glycosaminoglycans of the glycocalyx are critical to this process. The intact glycocalyx also promotes the production of prostacyclin, a potent inhibitor of platelet aggregation and leukocyte adhesion. Consequently, glycocalyx degradation removes this regulatory influence, favoring platelet adhesion, activation, and microthrombosis, further contributing to the ischemic burden in DCI (69).

The glycocalyx is also integral to the endothelial production of NO, a critical mediator of vascular tone and neurovascular coupling. Mechanical forces such as shear stress activate eNOS, leading to NO synthesis and subsequent smooth muscle cell relaxation. This mechanism ensures the dynamic regulation of cerebral blood flow according to local metabolic demands. Following aSAH, impairment of the NO pathway, whether through decreased eNOS activity, NO scavenging by hemoglobin breakdown products, or oxidative inactivation of NO, contributes to microvascular vasoconstriction and neurovascular uncoupling. These alterations lead to impaired oxygen and nutrient delivery, regional hypoperfusion, and the subsequent development of DCI (10, 23, 67).

Moreover, the glycocalyx contributes to oxidative homeostasis by housing antioxidants that neutralize ROS. Damage to the glycocalyx not only compromises mechanical barrier function but also diminishes antioxidative defences, resulting in excessive ROS accumulation. Increased ROS levels further exacerbate vascular injury by uncoupling eNOS, promoting apoptosis and autophagy, and reducing NO bioavailability. Thus, glycocalyx disruption represents a central event linking endothelial dysfunction, oxidative stress, microthrombosis, and impaired vasoregulation in the complex pathophysiology of DCI (31, 67, 70).

### Thrombo-inflammation

Clinical and experimental evidence strongly suggests that formation of microthrombi plays a crucial role in the pathophysiology of DCI (32, 71–74). Autopsy studies by Stein et al. revealed a strong correlation between the burden of microthrombi in various brain regions and the incidence of DCI in patients who died from aSAH (71).

Following aSAH, the accumulation of blood in the subarachnoid space creates a procoagulant and proinflammatory environment, triggering a cascade of pathological events. aSAH, in particular, causes significant vascular injury, leading to the activation of procoagulant mechanisms and platelet activation. There is growing evidence that platelets and platelet activation play a significant role in microthrombus formation and DCI development. Activated platelets release procoagulant factors, perpetuating a self-reinforcing cycle of coagulation and microthrombus formation. Endothelial injury and tissue ischemia further exacerbate microthrombus formation through the production of ROS and inflammatory mediators, a process known as thrombo-inflammation. Thrombo-inflammation, a major driver of DCI, involves the interplay between coagulation and inflammation, with various feedforward mechanisms reinforcing the interaction (43, 72). Cell adhesion molecules (CAM), particularly P-selectin, play a central role in thrombo-inflammation (17, 43, 73, 74). Following aSAH, the expression of CAMs such as P-selectin, E-selectin, ICAM-1, and VCAM-1 is upregulated on the endothelial surface, facilitating the recruitment of inflammatory cells (75–77). These recruited macrophages and neutrophils enter the subarachnoid space, where they initiate inflammatory pathways through the secretion of cytokines and chemokines. The phagocytosis of red blood cells and the subsequent cell death of infiltrating leukocytes generate a plethora of toxic molecules that further potentiate procoagulant and proinflammatory pathways (43).

P-selectin interacts with the leukocyte ligand PSGL-1, and increased circulating PSGL-1 expression has been independently associated with the occurrence of DCI and worse outcomes at six months (Barthel index score) (23, 78). Ishikawa et al. provided the first evidence that prothrombogenic and inflammatory responses are induced in the brain following aSAH. Their study observed interactions between platelets, leukocytes, and endothelial cells in postcapillary venules on the cerebral surface after experimental subarachnoid hemorrhage, demonstrating early platelet adhesion to the vessel wall, followed by leukocyte recruitment and platelet-leukocyte adhesion. They also demonstrated that P-selectin mediated these interactions. Platelets expressing P-selectin can interact with leukocytes expressing PSGL-1 or with endothelial PSGL-1 ligands. Blocking P-selectin with a specific antibody attenuated leukocyte rolling and adhesion. Additionally, non-P-selectin interactions between platelets and the endothelium, involving glycoprotein IIb/IIIa binding to endothelial ICAM-1 and fibrinogen cross-linking, were described (74).

Transient global hypoperfusion after aSAH initiates a cascade of events leading to EBI, including the activation of inflammatory cascades and the upregulation of endothelial adhesion molecules. Frontera et al. observed that patients with severe brain injury following aSAH suffered from significantly elevated platelet activation compared to less severely affected patients and controls. As early brain injury worsened, platelet activation and inflammation increased. These early events likely create the foundation upon which DCI develops in a continuum (42). Patients with impaired platelet function, resulting from intake of acetylsalicylic acid, as assessed by *in vitro* platelet function testing (PFA), showed a lower rate of DCI versus patients with regular tested primary hemostasis, although this did not correlate with better outcomes as measured by the modified GOS (79).

Kumar et al. demonstrated that patients with aSAH had significantly elevated levels of VWF and significantly lower plasma levels of a disintegrin and metalloproteinase with a thrombospondin type 1 motif, member 13 (ADAMTS-13). The lower ratio of plasma ADAMTS-13 activity to VWF antigen in these patients suggests a relative deficiency of ADAMTS-13 (80).

VWF, a multimeric protein crucial in hemostasis, facilitates platelet binding to the endothelium and platelet aggregation (81). Ultra-large VWF multimers, released from endothelial cells (Weibel-Palade bodies) following inflammatory stimulation, are highly thrombogenic, forming string-like structures that recruit platelets to the site of injury. ADAMTS-13 regulates VWF function by cleaving it at the central A2 domain, thereby reducing its prothrombogenic potential and repressing thrombosis-induced inflammation through downregulation of P-selectin (17, 80, 81).

Patients with DCI after aSAH exhibited a more significant decrease in ADAMTS-13 compared to those without DCI (82). Increased platelet activation, characterized by elevated levels of platelet-derived thromboxane B2, *β*-thromboglobulin, platelet-activating factor, and P-selectin, along with decreased ADAMTS-13 and elevated VWF antigen and activity, further support the role of platelet-mediated thrombosis in the development of DCI (34, 43, 80–84).

Next to promoting microthrombus formation, platelets play a critical role in the development of DCI by amplifying the neuro-inflammatory response through the secretion of proinflammatory mediators leading to leukocyte recruitment and triggering cortical spreading depolarizations through the release of glutamate (34). Given the importance of thrombo-inflammation in DCI and the propensity of aSAH patients to develop thromboembolic events, the role of viscoelastic tests such as thrombo-elastography (TEG) and rotational thrombo-elastometry (ROTEM) have been investigated in these patients. Importantly, the anticipated hypercoagulable status in these patients is not always reflected by standard coagulation tests (e.g., prothrombin time, activated partial thromboplastin time, D-dimer). More comprehensive coagulation profiles can be obtained using viscoelastic tests. A systematic review by Tjerkstra et al. confirmed a hypercoagulable state in aSAH patients, as evidenced by TEG and ROTEM-parameters of increased clot strength, which were associated with DCI. The observed increased clot firmness appears to be due to fibrinogen and platelet activity, both of which are key elements of acute inflammation (85). Interestingly, enhanced fibrinolysis was not detected, which may explain why antifibrinolytic therapies like tranexamic acid have no impact on mortality, poor outcomes, or DCI in aSAH patients, despite the reduced risk of rebleeding (86).

This state of hypercoagulability observed in patients following aSAH has been corroborated by recent findings. In a study conducted by Raatikainen et al., rotational thromboelastometry parameters (EXTEM and FIBTEM) and D-dimer levels were analyzed at four time-points after the hemorrhage. While increased blood coagulation was not linked to DCI, a significantly shorter EXTEM clot formation time (EXTEM-CFT) was observed on post-bleed days 4-5 and 7-8 in patients with poor neurological outcomes, as assessed by the extended GOS. Additionally, FIBTEM maximum clot firmness (FIBTEM-MCF) was significantly elevated in these patients on post-bleed days 4-5, 7-8, and 11-12. Moreover, patients with unfavorable neurological outcomes exhibited consistently higher D-dimer levels across all analyzed time points (87). We emphasized the important role of platelets in mediating hypercoagulability following aSAH. Indeed, this hypercoagulability is initiated following ictus through endothelial injury and subsequently driven by platelet activation in the thrombo-inflammatory process. However, the pathophysiology of this hypercoagulation is more complex and the pivotal role of leukocytes should be emphasized as well. Immune system activation exerts a significant influence on blood coagulation and the pathological formation of thrombi. Leukocytes, including monocytes, macrophages, and neutrophils, express and release both coagulation and fibrinolytic factors, while engaging with the hemostatic system through innate immune mechanisms. These cells actively regulate the coagulation cascade, modulate the hemostatic functions of endothelial cells and platelets, and contribute to microvascular thrombosis. Additionally, leukocytes influence fibrinolysis, facilitate thrombus resolution, and mediate the clearance of coagulation factors via phagocytosis (88). Under physiological conditions, systemic leukocytes help maintain anticoagulation by expressing various anticoagulant factors, such as endothelial protein C receptor, TFPI, and thrombomodulin. However, upon activation of proinflammatory signaling pathways, leukocytes undergo phenotypic changes that enhance coagulation. This occurs through the release of procoagulant factors and the downregulation or degradation of anticoagulant factors. Furthermore, leukocyte surfaces serve as a platform for coagulation factor assembly. Monocytes, the primary intravascular source of tissue factor (TF), exhibit increased TF activity and TF-induced thrombin generation in response to inflammatory stimuli (88). As mentioned earlier, neutrophils release MMP and serine proteases that promote coagulation activation through different mechanisms (activation of FV, FVIII, FX, and degradation of antithrombin, TFPI, and heparin cofactor II). The role of NETosis, and its contribution in coagulation activation, was discussed earlier (88). While leukocytes are essential for initiating and sustaining inflammation, they also contribute to a hypercoagulable state following aSAH, playing a central role in the process of thrombo-inflammation (43, 88, 89). Recent evidence suggests a potential pivotal role for TF in initiating coagulation in aSAH, driven by overactivation of the NLRP3 inflammasome. The NLRP3 inflammasome acts as upstream regulator of inflammation pathways in various inflammatory conditions. It recruits CASP1, which cleaves the proinflammatory cytokines IL-1β and IL-18, and induces pyroptosis – an inflammatory form of programmed cell death characterized by increased plasma membrane permeability. Pyroptotic macrophages release TF, a critical initiator of coagulation cascades. In aSAH patients, the NLRP3 inflammasome, as a master regulator of sterile inflammation, may drive coagulation and thrombosis through pyroptosis, releasing proinflammatory cytokines and tissue factor (90). In their study, Díaz-García et al. demonstrated that patients with aSAH exhibit overactivation of the NLRP3 inflammasome, evidenced by increased expression of NLRP3 and CASP1 in monocytes, along with elevated levels of inflammasome end-products, including IL-1β, IL-18, GSDMD, and TF (90). Serum TF levels were positively correlated with the Acute Physiology and Chronic Health Evaluation II (APACHE II) and WFNS scores and negatively correlated with the Glasgow Coma Scale. Furthermore, TF levels were significantly higher in patients with poor outcomes compared to those with favorable outcomes. The role of TF as a prognostic biomarker for complications in aSAH warrants further investigation (90).

Several studies have explored strategies to mitigate DCI by targeting coagulation and platelet aggregation. Systemic anticoagulation with enoxaparin has demonstrated efficacy in reducing DCI in patients with low-grade aSAH (Hunt and Hess grades I-III); however, this effect was not observed in the general study population, when cases of more severe bleeding were taken into account (91). The use of nadroparin for prevention of DCI is currently under investigation in a phase 2 trial (NCT04507178). Three retrospective studies have reported a beneficial impact of low-dose heparin on both DCI incidence and functional outcomes (92–94). Notably, the results of the ASTROH trial (NCT02501434) are highly anticipated. This multi-center RCT aims to assess the safety and clinical efficacy of continuous low-dose intravenous unfractionated heparin infusion for the prevention of aSAH-induced neurocognitive dysfunction and other delayed neurological deficits.

In addition to anticoagulants, antiplatelet agents such as aspirin, dipyridamole, and ticlopidine have been investigated for their potential role in DCI prevention. A meta-analysis suggested a modest trend toward improved outcomes, although this was accompanied by a possible increase in hemorrhagic complications. Tirofiban, a glycoprotein IIb/IIIa inhibitor, is currently being evaluated in the iSPASM trial (NCT03691727) to further elucidate its therapeutic potential in this context (95).

### Neuro-inflammation

As previously stated, neuro-inflammation is an emerging concept that is being assigned an increasingly important role in the pathophysiology of both EBI and DCI (4, 32).

The inflammatory processes triggered during EBI play a significant role in the development of DCI. Following aSAH, a robust systemic and localized inflammatory response occurs and leads to the activation and recruitment of immune cells at the site of injury (17, 31). The intensity of this inflammatory response is predictive of DCI and adverse outcomes, prompting a growing focus on the cellular and molecular mechanisms underlying aSAH induced neuro-inflammation.

DAMPs are a heterogenous group of molecules originating from various cellular compartments and released during cell injury or stress. These DAMPs are recognized by pattern recognition receptors (PRRs) such as TLR-2, TLR-4, and IL-1R on innate immune cells, leading to activation of inflammatory signaling pathways (e.g., NFκB pathway) and upregulated expression of multiple genes with augmented transcription and release of proinflammatory cytokines (10, 96).

Various DAMPs and PRRs have been identified as key players in neuro-inflammation and DCI. Notable DAMPs include High Mobility Group Box 1 (HMGB1), which interacts with TLR-2, TLR-4, TLR-9, and Receptor for Advanced Glycation End products (RAGE); S-100 proteins, which engage TLR-4 and RAGE; and fibronectin, heparan sulphate, and mitochondrial DNA (mtDNA), which interact with TLR-2, TLR-4, and TLR-9, respectively (96).

Hemoglobin degradation products, such as haem, methaemoglobin, and oxyhemoglobin, are particularly significant DAMPs that interact with TLR-4, leading to neuro-inflammation and contributing to DCI. HMGB1, a prototypical DAMP protein, functions as a non-histone DNA-binding nuclear transcription factor. Upon release from necrotic cells, HMGB1 triggers neuro-inflammation through interactions with TLR-2, TLR-4, TLR-9, and RAGE. Several studies have highlighted the role of HMGB1 in the pathophysiology of DCI, with elevated CSF levels of HMGB1 being associated with poor clinical outcomes, higher Hunt & Hess grades, and increased disability and dependence following aSAH (97–99).

Elevated systemic HMGB1 levels have also been linked to cerebral vasospasm, poor functional outcomes, and increased mortality (100). S100B, a small intracellular calcium-binding protein primarily expressed by astrocytes, is recognized for its prognostic value central nervous system pathologies (96). At elevated levels, S100B acts as a DAMP, exerting neurotoxic effects through interaction with RAGE. This can lead to neuronal death and the expression of proinflammatory cytokines. Following aSAH, increased S100B levels correlate with the severity of illness and serve as a predictor of outcomes (96).

The primary trigger for neuro-inflammation following aSAH is likely the extravasated blood and its degradation products, including haem, methaemoglobin, oxyhemoglobin and hemin. These interact with TLR-4 and, through the activation of myeloid differentiation primary response protein 88 (MyD88), NFkB and mitogen activated protein kinase (MAPK) signaling pathways, result in increased expression of TNFα and other proinflammatory genes (10, 17, 96). Since blood degradation products are water-soluble, they can cause widespread TLR-4 activation far from the initial site of injury. Additionally, extracellular DNA and DNA-binding proteins can act as DAMPs, serving as surfaces for the assembly of coagulation factor complexes, while histones activate platelets and stimulate thrombin generation (88, 101).

The activation and binding of neutrophils to extracellular DNA leads to the formation of NETs, which contributes to excessive thrombosis. These NETs contain neutrophil elastase, which further degrades anticoagulants. Haem, in addition to interacting with TLR-4, also promotes NET formation, thereby exacerbating neuro-inflammation (96).

The brain’s initial response to aSAH is predominantly mediated by resident microglia, the central nervous system’s primary phagocytes (17, 47). Upon activation by hemorrhage or other threats, microglia upregulate inflammatory cytokines, thereby amplifying neuro-inflammatory pathways (84). Research by Hanafy and Schneider highlights the critical role of microglia in DCI, demonstrating that microglial depletion significantly reduces neuronal cell death following aSAH (102, 103). Microglia are involved in various aspects of DCI, including neuro-inflammation, vasospasm, microthrombosis, and cortical spreading depolarizations (47).

Microglia contribute to sustained neuro-inflammation and vasospasm through the release of proinflammatory cytokines like TNFα, which promotes vasoconstriction (47). They are also implicated in microthrombosis, with thrombin-induced calcium release activating microglia. Additionally, microglia may influence cerebral blood flow through P2RY12 signaling, although this role has not been fully explored in aSAH models (47). Evidence suggests that CSDs induce a spreading calcium wave in microglia, potentially linking CSDs to microglial activation and subsequent neurotoxicity (89).

TLR-4, a PRR highly expressed in microglia, plays a crucial role in initiating neuro-inflammation following aSAH. The upregulation of TLR-4 expression after aSAH enables microglia to act as first responders by interacting with various DAMPs (10, 17, 96).

As previously discussed, there is a strong interplay between inflammation and coagulation. During the sustained microthrombus formation following aSAH, continued activation of coagulation by endothelial cells and platelets promotes activation of circulating leukocytes through protease activated receptor-1 (PAR-1) and TLR-4 (43, 88, 89). The binding of thrombin and other activated coagulant proteases to specific PARs, and the binding of fibrin to TLR-4 on inflammatory cells will further affect inflammation through release of proinflammatory cytokines and chemokines, which further modulates coagulation (101).

Various blood coagulation factors and regulatory proteins of the coagulation cascade have been implicated in influencing the pathophysiology of central nervous system diseases (Amyotrophic Lateral Sclerosis, Alzheimer’s disease, Parkinson disease, multiple sclerosis, and ischemic stroke and post-ischemic epilepsy). Among these, the protease-activated receptor pathway serves as a central regulatory hub, primarily mediated by thrombin and aPC. These proteins extend their influence beyond coagulation, impacting synaptic homeostasis by modulating neuronal networks (104). Their effects are exerted on resident CNS cells, including neurons, astrocytes, and microglia, as well as on circulating immune cells and the extracellular matrix. Thrombin exerts a wide range of effects on the CNS cells, including neurite retraction, morphological alterations, and astrocyte and microglial mitosis. The impact of thrombin on astrocytes is dose-dependent: low concentrations promote cell survival and synaptic plasticity, whereas at high thrombin concentrations, reactive astrocytes and microglia proliferate and lose their glutamate regulatory function, leading to excitotoxicity and production of reactive oxygen species, inflammatory cytokines (IL-1 *β*, TNF-*α*), and inducible NOS. Neurons suffer neurite retraction, intracellular Ca^2+^ upregulation, and eventually cell death (104). The BBB is damaged and the extracellular matrix is digested by different MMPs.

Preconditioning with low doses of thrombin has been shown to attenuate the inflammatory response and prevent cell death in ischemic and hemorrhagic stroke models. These outcomes are mediated through PAR activation. Specifically, PAR-1 activated by thrombin interacts with various G proteins, driving inflammatory processes, upregulating cytokines, and increasing cell adhesion molecule expression. The diverse intracellular signaling pathways involved underpin the pleiotropic activities of PARs. Thrombin fully cleaves PAR, leading to its internalization (104).

In ischemic stroke, elevated thrombin levels have been linked to changes in synaptic transmission, reduced excitability, and the potential development of post-ischemic seizures (resulting from an impairment of the aminobutyric acid (GABA)-dependent inhibitory tone). In contrast, aPC exerts different effects on PARs. Together with its co-receptor endothelial protein C receptor, aPC binds to PARs, promoting pathways that enhance cell viability and reduce cell death in inflammatory models. While thrombin triggers RhoA activation, leading to endothelial dysfunction, aPC activates Rac1, which is essential for maintaining endothelial stability. Unlike thrombin, aPC only partially cleaves PAR, avoiding its degradation. Through PAR-1 activation, aPC supports the neurovascular unit, stabilizing the BBB and mitigating inflammation (104).

The neuroprotective role of aPC in aSAH has been explored using a rat model. Followed experimental subarachnoid hemorrhage, aPC expression significantly decreased while the expression of the NLRP3 inflammasome increased over time. NLRP3 activation is closely associated with inflammatory injury and neurological dysfunction in aSAH, and its induced pyroptosis contributes to neuroinflammatory damage. In the rat model, treatment with recombinant aPC improved neurological function, inhibited pyroptosis in a dose-dependent manner, and alleviated EBI caused by aSAH by suppressing NLRP3 inflammasome activation (105, 106).

It is important to note that microglia, while playing a central role as resident immune cells of the CNS, are not the sole contributors to the immune response following aSAH (17). Peripheral immune responses are also significant. Following aSAH, the increased expression of CAMs (e.g., ICAM-1) and extracellular MMPs (e.g., MMP-9) leads to BBB disruption, allowing peripheral immune cells to infiltrate the subarachnoid space and brain parenchyma (10, 67). Even without directly entering the CNS, peripheral leukocytes can influence the CNS immune response through the secretion of cytokines. Circulating immune cells also serve as a critical link between vascular dysfunction, vascular inflammation, and the systemic immune response (17).

### The glymphatic system

The glymphatic system is a brain-wide network of lymphatic vessels that line the dural sinuses. Astrocytes, along with their water channel protein AQP4, play a pivotal role in regulating glymphatic function by surrounding these lymphatic vessels (107, 108). Accumulation of red blood cell degradation products within the subarachnoid space triggers a range of neuroinflammatory responses, and dysfunction of the glymphatic system may contribute to the pathogenesis of DCI and neuro-inflammation. Endothelial cells within the lymphatic system facilitate the transport of CSF, macromolecules, and immune cells from the CSF to the cervical lymph nodes, playing a key role in clearing metabolic waste products and modulating the immune response (17, 84, 107, 108). Following aSAH, blood components, toxic metabolites, and reactive lymphocytes enter the brain parenchyma through paravascular pathways, triggering extensive neuro-inflammation in perivascular regions. This inflammation is characterized by robust microglial and astrocyte activation, with increased expression of TLR-4 and TNF-*α* (108). The ensuing inflammatory milieu can cause microvascular spasm, disrupting CSF flow and cerebrovascular dynamics. As a result, congestion in the perivascular space may predispose adjacent brain tissue to ischemia and exacerbate DCI (23, 31).

Emerging evidence highlights the critical role of AQP4 in maintaining glymphatic flow, which is often impaired following aSAH (17). AQP4, highly expressed in astrocytic end-feet, facilitates the transport of interstitial fluid (ISF) through the glymphatic system. After aSAH, AQP4 dysfunction occurs, often accompanied by upregulation of its expression (23, 108). In AQP4 knockout rats (AQP4 -/-), neuron loss in the hippocampus and disruption of the BBB following aSAH were markedly worsened. In these knockout models, both CSF inflow and ISF outflow are significantly obstructed after aSAH. Beyond its role in reducing brain edema, AQP4 is essential for facilitating the exchange between CSF and ISF, thereby mitigating the toxic accumulation of metabolic waste products that can lead to neuronal injury and BBB disruption (109–111).

**Figure 3:**
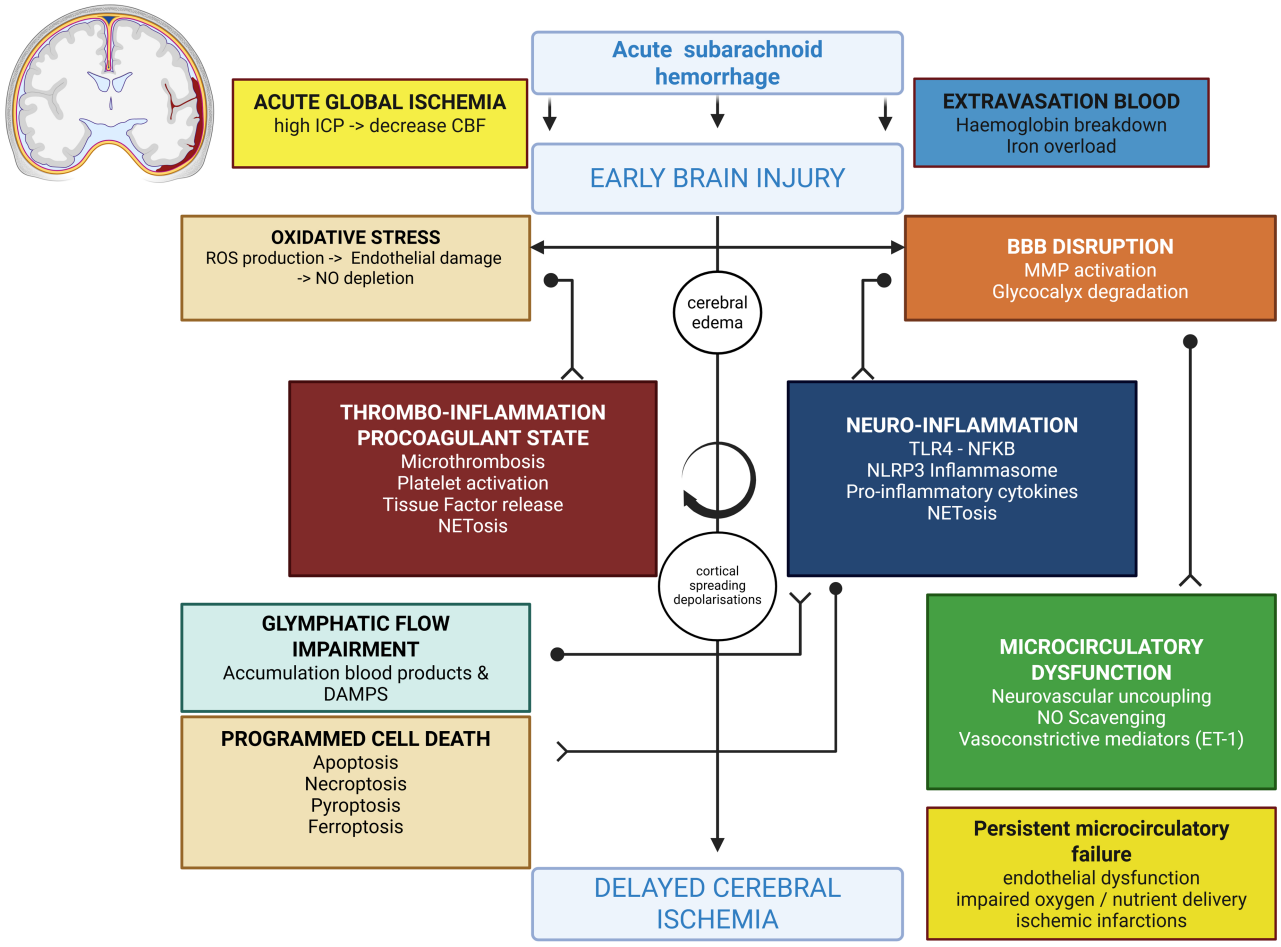
Overview of the main pathophysiological mechanisms of EBI and DCI. Created in BioRender. Renette, W. (2025) https://BioRender.com/wxxq4ff.

Additionally, these processes are compounded by the obstruction of lymphatic flow due to microthrombi formation in the paravascular space. The potential role of microthrombi in impeding CSF flow and contributing to EBI is supported by evidence from subarachnoid hemorrhage animal models, where an early reduction in cortical perfusion, both dependent and independent of intracranial pressure, has been observed. Intracisternal administration of tissue plasminogen activator (tPA) increased cortical blood volume, reduced ICP, and partially restored CSF flow in mice within 24 h (112). Subsequent studies have confirmed these findings, demonstrating that subarachnoid hemorrhage animals treated with tPA showed significant neurological improvement and reduced neuro-inflammation seven days post-hemorrhage (108). Glymphatic flow obstruction also induces retrograde flow in penetrating arteries, which can lead to vasoconstriction and prolonged vascular spasm. This may initiate CSD and further aggravate DCI (23) (Figure 3; Table 1).

**Table 1:** Overview of potential biomarkers of EBI and DCI following aSAH.

Biomarkers of EBI/DCI following aSAH		
Endothelial Dysfunction	Asymmetric Dimethylarginine (ADMA): Endogenous NOS inhibitor Endothelin-1: vasoconstrictor elevated when NO impairment Soluble adhesion molecules ICAM-1, VCAM-1, E-selectin: markers of endothelial activation Syndecan 1: marker of glycocalyx degradation von Willebrand Factor: released from damaged endothelium	(32, 66, 113–115)
Cortical Spreading Depolarizations	Glutamate: excitotoxicity marker, via microdialysis Lactate/Pyruvate ratio: metabolic distress Direct Electrocorticography detection: method Neuron-Specific Enolase: nonspecific, indicator of neuronal stress	(34, 116, 117)
Disruption of Blood–Brain Barrier	S100B: astrocytic protein leaking into blood when disruption of BBB, serum MMP-9: degrades tight junctions and basal lamina Aquaporin-4: expression changes	(10, 32, 118, 119)
Cerebral Edema	Glial Fibrillary Acidic Protein (GFAP): astrocytic injury marker Aquaporin-4 (AQP4): key role in edema formation Interleukin-6: edema-related inflammation mediator Brain Natriuretic Peptide (BNP)	(32, 119–125)
Neuro-inflammation	Myeloperoxidase (MPO): part of NETs, activated neutrophils Cell-free DNA (cfDNA): NETs/necrotic cells Citrullinated Histone H3 (Cit-H3): specific marker of NETosis IL-1β, IL-6, TNF-*α*: pro-inflammatory cytokines High-Mobility Group Box 1 (HMGB1): DAMP, released during inflammation	(10, 126–128)
Thrombo-inflammation	Tissue factor PSGL-1 FIBTEM (thrombo-elastometry) ADAMTS13 – VWF	(78, 85, 87, 90)
Programmed Cell Death	*Ferroptosis biomarkers* Malondialdehyde (MDA): lipid peroxidation marker 4-Hydroxynonenal (4-HNE): lipid peroxidation byproduct Glutathione depletion/GPX4 inactivation: GPX4 downregulation drives ferroptosis Increased free Iron (Fe^2+^): catalyzation of lipid ROS generation *Pyroptosis biomarkers:* Gasdermin D (GSDMD) cleavage fragments: executioner of pyroptosis Caspase-1 activity: central to inflammasome activation IL-1β and IL-18: cytokines released during pyroptosis	(50, 51, 129)

## Therapeutic implications and future directions

As previously stated, the integrity of the BBB is compromised during EBI, with multiple underlying pathophysiological mechanisms, including MMP-9 activity, being identified. Despite the critical role of BBB disruption in disease progression, clinical studies investigating its implications remain scarce (130). In addition, the role of NETosis in microthrombus formation, microvascular spasms, and subsequent microcirculatory dysfunction has recently gained increased attention. Targeting the reduction of NETs thus warrants investigation as a potential therapeutic strategy. Furthermore, oxidative stress cascades involving ROS, neuroinflammatory pathways such as TLR-4, NFκB, and NLRP3 inflammasome-driven pyroptosis, vascular endothelial growth factor signaling, as well as diverse forms of cell death, including organellar dysfunction, autophagy, apoptosis, ferroptosis, and necroptosis, require further research to assess their potential as effective therapeutic targets (130).

### Targeting NETosis

NETs have been increasingly implicated in the development of microvascular occlusion, endothelial dysfunction, and microvasospasms following aSAH. Experimental models have demonstrated that enzymatic degradation of NETs or inhibition of NETosis, such as through PAD4 inhibitors, can attenuate microvasospasms and microthrombosis. Pharmacologic interventions aimed at preventing NET formation or promoting NET degradation may thus represent promising strategies to protect the cerebral microcirculation and reduce the incidence of DCI (128).

### Inhibition of the NLRP3 inflammasome

The NLRP3 inflammasome plays a central role in sterile neuro-inflammation. Its activation promotes the release of proinflammatory cytokines such as 1 L-1β and IL-18 and induces pyroptosis, leading to endothelial injury and TF release, thereby fueling thrombo-inflammation. Overactivation of NLRP3 inflammasome has been correlated with worse clinical outcomes, making it an attractive therapeutic target. Inhibition of NLRP3 activation or downstream signaling pathways holds promise for reducing neuro-inflammation, preserving endothelial integrity, and mitigating DCI. The beneficial effects of dexmedetomidine in patients with aSAH are recently published and were associated with significantly lower in-hospital mortality compared to propofol and midazolam (131, 132). Dexmedetomidine exerts this neuroprotective effect possibly by suppressing the inflammatory response through interaction with the TLR4/NFκB pathway and the NLRP3 inflammasome (133–135).

### Activated protein C as a neuroprotective agent

aPC exerts potent anti-inflammatory, anticoagulant, and cytoprotective effects. Following aSAH, endogenous aPC activity is diminished, contributing to a hypercoagulable, proinflammatory state. aPC can modulate the inflammatory response by inhibiting NLRP3 inflammasome activation, preserving endothelial barrier function, and mitigating apoptosis through PAR-1 signaling. In experimental models, administration of recombinant aPC improved neurological outcomes and reduced neuroinflammation. Future clinical trials are warranted to evaluate the potential of aPC-based therapies in modulating both inflammation and thrombosis following aSAH.

### Glycocalyx repair and endothelial protection

The endothelial glycocalyx is crucial for maintaining vascular integrity, and its degradation after aSAH promotes inflammation, microthrombosis, and impaired NO production. Emerging evidence suggests that glycocalyx shedding may precede DCI, making its preservation or restoration a promising therapeutic target (67). Sphingosine-1-phosphate (S1P), by inhibiting MMPs and promoting glycocalyx synthesis, has shown protective effects in preclinical models. Similarly, albumin infusion, a major source of S1P, has been associated with restored glycocalyx thickness and improved microvascular stability (67). The HASH trial will investigate the use of human albumin, compared to standard fluid therapy, in patients with aSAH (136). Other approaches, including MMP inhibitors (e.g., batimastat) and glucocorticoids such as dexamethasone, have demonstrated the potential to protect or regenerate the glycocalyx and reduce BBB disruption (67). Although several pharmacologic strategies, including sulodexide, metformin, and antithrombin, have shown preliminary promise, clinical evidence remains limited (67). Future studies are needed to determine whether glycocalyx-targeted therapies can effectively prevent or treat DCI, and to establish glycocalyx integrity as a potential biomarker of disease progression.

### Anti-inflammatory therapies

Despite the central role of inflammation in EBI and DCI, anti-inflammatory therapies have yet to yield consistent clinical benefits. Corticosteroids, NSAIDs, thromboxane inhibitors, cyclosporine, statins, and monoclonal antibodies have shown limited success, partly due to nonspecific immunosuppression. A study investigating the prophylactic treatment with etanercept, a TNFα blocker, was withdrawn (NCT01865630). The FINISHER trial, a multicenter placebo-controlled Phase III RCT, investigates the effect of dexamethasone on functional outcome (modified Rankin Scale at 6 months) (137). Also the efficacy of deferoxamine, a hydrophilic chelator of iron that prevents formation of iron related free radicals, is investigated (NCT04566991). Newer approaches aim to target inflammation more selectively, focusing on TLR signaling inhibition, blockade of HMGB1 protein, or neutralization of DAMPs. Restoration of glymphatic clearance mechanisms also holds potential as an adjunctive strategy to reduce neuro-inflammation. Further research is needed to address the challenges of systemic and neuro-inflammation in aSAH (10, 17) (Table 2).

**Table 2:** Overview of trials aimed at DCI prevention and management.

Delayed cerebral ischemia: overview of trials				
Trial name	Treatment	Targeted mechanism	Outcome/Status	References
CONSCIOUS-1	Clazosentan (endothelin receptor antagonist)	Endothelial dysfunction/vasospasm	Reduces vasospasm, no outcome improvement	(55)
CONSCIOUS-2	Clazosentan, post surgical clipping	Endothelial dysfunction/vasospasm	No significant effect on mortality/morbidity	(56)
CONSCIOUS-3	Clazosentan, post endovascular treatment	Endothelial dysfunction/vasospasm	Reduced vasospasm-related morbidity, no outcome gain	(57)
Endo et al.	Clazosentan post endovascular or surgical treatment (2 prospective RCTs)	Endothelial dysfunction/vasospasm	Reduced vasospasm-related morbidity, reduced all-cause mortality	(59)
REACT trial	Clazosentan	Endothelial dysfunction/vasospasm	No benefit on clinical deterioration due to DCI	(60, 61)
Simard et al. (2013)	Low-dose IV heparin versus subcutaneous heparin Retrospective	Thrombo-inflammation/platelet activation	IV heparin safe, less clinical vasospasm	(94)
James et al. (2019)	Low-dose IV heparin Retrospective	Thrombo-inflammation/platelet activation	Improved cognitive outcome	(93)
Kole et al. (2021)	Low-dose IV heparin versus standard subcutaneous heparin Retrospective	Thrombo-inflammation/platelet activation	Less delayed neurological deficits in low-dose IV heparin group	(92)
ASTROH trial NCT02501434	Low-dose unfractionated heparin	Thrombo-inflammation/platelet activation	Ongoing	
Nadroparine NCT04507178	Nadroparin (LMWH)	Thrombo-inflammation/Platelet activation	Ongoing	
iSPASM trial (NCT03691727)	Tirofiban (GPIIb/IIIa inhibitor)	Thrombo-inflammation/Platelet inhibition	Ongoing	
Etanercept trial (NCT01865630)	Etanercept (TNFα blocker)	Neuroinflammation	Withdrawn	
FINISHER trial	Dexamethasone	Neuro-inflammation	Ongoing	
Deferoxamine trial (NCT04566991)	Deferoxamine (iron chelator)	Oxidative stress/Neuroinflammation	Ongoing	
CLASH	Eculizumab Complement C5 antibody (primary outcome: C5a concentration CSF day 3 after ictus)	Neuro-inflammation	No benefit	(138)

## Conclusion

The early phases following aSAH involve a complex interplay of systemic and cerebral factors that critically determine clinical outcomes. Acute increases in ICP trigger transient global ischemia, further exacerbated by intracerebral hemorrhage and brain edema. This is accompanied by a surge of catecholamines, leading to endothelial dysfunction and contributing to both EBI and DCI. EBI is a major determinant of clinical prognosis, driven by multiple mechanisms including cerebral metabolic distress and oxidative stress, CSDs, neuro-inflammation, microvascular dysfunction, BBB disruption, cerebral edema, and cellular apoptosis. The processes are highly interconnected, collectively worsening neuronal cell death and dysfunction, highlighting the importance of EBI in influencing long-term aSAH outcomes.

Endothelial dysfunction and microvascular dysregulation, largely due to impairments in the NO-cGMP signaling pathway, play a central role in EBI. CSDs contribute to both EBI and DCI, while disruption of the BBB exacerbates EBI and is compounded by cerebral edema. Recognizing and addressing these mechanisms is vital for enhancing patient care after aSAH.

DCI, which affects 20-30% of aSAH survivors, remains a significant contributor to poor neurological outcomes. Although traditionally linked to cerebral vasospasm, recent evidence suggests a multifactorial etiology involving microcirculatory dysfunction, thrombo-inflammation, neuro-inflammation, and CSD. Nimodipine continues to be the most effective therapy for reducing morbidity and mortality post-aSAH, acting through mechanisms which extend beyond vasospasm mitigation.

Microcirculatory dysfunction, characterized by damage to the endothelial glycocalyx and impaired endothelial function, plays a key role in DCI by disrupting oxygen and nutrient delivery. Thrombo-inflammation, which involves platelet activation and microthrombus formation, is central to the pathogenesis of DCI. The resulting platelet-mediated thrombosis, coupled with neuroinflammatory processes, exacerbates neuronal injury and increases the risk of DCI. Next to platelet activation, TF release, through a process of NLRP3 inflammasome induced pyroptosis, is recognized as important driver of micro-thrombosis, in confluent with the emerging role of NETosis. Dysfunction of the glymphatic system, particularly involving AQP4, further complicates DCI by impairing CSF clearance and amplifying neuro-inflammation. Neuro-inflammation, driven by DAMPs and peripheral immune responses, perpetuates neuronal injury and worsens DCI outcomes.

A thorough understanding of the interrelated pathophysiological mechanisms is crucial for developing effective therapeutic strategies to mitigate DCI and improve post-SAH outcomes. Further research into targeted anti-inflammatory therapies, as well as therapies aiming other pathophysiological mechanisms is needed to comprehensively address this complex and multifaceted challenge.
